# Efficacy and safety of topical 0.1% cannabidiol for managing recurrent aphthous ulcers: a randomized controlled trial

**DOI:** 10.1186/s12906-023-03886-0

**Published:** 2023-02-20

**Authors:** Chalapinyo Umpreecha, Kanokporn Bhalang, Dusadee Charnvanich, Jittima Luckanagul

**Affiliations:** 1 Pathum Thani Provincial Public Health Office, Ministry of Public Health, 14 Rat Amnuay Road, Bang Prok, Muang, Pathum Thani, 12000 Thailand; 2 Department of Oral Medicine, Faculty of Dentistry, Chulalongkorn University, 34 Henri-Dunant Road, Wangmai, Pathumwan, Bangkok, 10330 Thailand; 3 Department of Pharmaceutics and Industrial Pharmacy, Faculty of Pharmaceutical Sciences, Chulalongkorn University, 254 Phayathai Road, Wangmai, Pathumwan, Bangkok, 10330 Thailand

**Keywords:** Cannabidiol, CBD, Cannabis, Cannabinoids, Recurrent aphthous ulcer, Recurrent aphthous stomatitis, Topical steroids

## Abstract

**Background:**

Although topical steroids constitute the first-line therapy for recurrent aphthous ulcers (RAUs), their long-term use often leads to candidiasis. Although cannabidiol (CBD) can be an alternative for pharmacologically managing RAUs due to its analgesic and anti-inflammatory in vivo effects, there is a lack of clinical and safety trials concerning its use. The aim of this study was to evaluate the clinical safety and efficacy of topical 0.1% CBD for managing RAU.

**Methods:**

A CBD patch test was performed on 100 healthy subjects. CBD was applied on the normal oral mucosa of 50 healthy subjects 3 times/day for 7 days. Oral examination, vital signs, and blood tests were performed pre- and post-CBD use. Another 69 RAU subjects randomly received one of three topical interventions: 0.1% CBD, 0.1% triamcinolone acetonide (TA), or placebo. These were applied on the ulcers 3 times/day for 7 days. The ulcer and erythematous size were measured on day 0, 2, 5, and 7. Pain ratings were recorded daily. The subjects rated their satisfaction with the intervention and completed a quality-of-life questionnaire (OHIP-14).

**Results:**

None of the subjects exhibited allergic reactions or side effects. Their vital signs and blood parameters were stable before and after the 7-day CBD intervention. CBD and TA significantly reduced ulcer size more than placebo at all time points. The erythematous size reduction was higher in the CBD intervention than the placebo on day 2, while TA reduced the erythematous size at all time points. The pain score in the CBD group was lower compared with placebo on day 5, whereas TA reduced pain more than placebo on day 4, 5, and 7. The subjects receiving CBD reported higher satisfaction than placebo. However, the OHIP-14 scores were comparable among the interventions.

**Conclusions:**

Topical 0.1% CBD reduced ulcer size and accelerated ulcer healing without side effects. CBD exerted anti-inflammatory effects in the early stage and an analgesic effect in the late RAU stage. Thus, topical 0.1% CBD might be more appropriate for RAU patients who decline to take topical steroids, except for cases where CBD is contraindicated.

**Trial registration:**

Thai Clinical Trials Registry (TCTR) Number TCTR20220802004. Retrospectively registered on 02/08/2022.

## Background

Recurrent aphthous ulcer (RAU) is the most common painful oral lesion and affects patients’ quality of life (QoL) [1]. RAU is a self-limiting ulceration [2]. It is more common in females from childhood through adolescence. Despite having a high prevalence (~ 20% of the general population) [1], the precise etiology remains unclear [3]. However, RAU has been found to be associated with immunological disorders [4]. Other predisposing factors include trauma, nutritional deficiencies, food allergies, genetics, stress, microbial factors, hormonal defects, and underlying medical diseases (e.g., Crohn’s disease, Behcet’s disease, and anemia) [1, 5].

RAU presents as an ovoid or round well-defined ulcer, with a pseudomembranous yellowish gray center, and an erythematous circumscribed border [6]. Minor aphthous ulcer is the most common RAU type, affecting 80% of RAU patients. It typically presents as less than 1 cm in diameter [5] and spontaneously resolves in 4–14 days without scarring [7].

Currently, there are no curative therapies for RAU. Thus, RAU management primarily focuses on pain relief, reducing inflammation, and promoting wound healing. Topical steroids are an acceptable first-line pharmacological intervention for RAU [8, 9], however, these also have numerous side effects, particularly if the ulcers are recurrent and long-term steroid use is necessary. Steroids suppress the immunological response, which can lead to oral candidiasis and other infections [10]. Natural substances such as myrtle, quercetin, and Damask rose have been suggested as alternative treatments for RAU due to their analgesic, wound healing-promoting, and anti-inflammatory effects, respectively [9].

In recent years, there is considerable public interest in the use of cannabis for medical purposes. Cannabinoids are one of the major medicinal components of cannabis. They are synthesized in the human body and produced by the cannabis plant. The two active medical components from the cannabis plant are Δ^9^‐tetrahydrocannabinol (THC) and cannabidiol (CBD) [11]. THC has several medicinal effects, including being psychoactive. In contrast, CBD is non-psychoactive and has meaningful analgesic, anti-inflammatory, and anti-convulsant effects [12]. Furthermore, CBD suppresses pro-inflammatory cytokine expression [13, 14]. Inflammation plays a crucial role in the wound healing process [15]. However, excessive inflammatory mediator levels can delay healing [16, 17]. Therefore, reducing pro-inflammatory cytokine expression optimizes healing time and reduces pain intensity [17]. Thus, CBD may promote wound healing due to its anti-inflammatory effects. Altogether, the CBD reported in vivo effects, such as pain relief, anti-inflammation, and promoting wound healing [18], would be beneficial in clinically managing painful inflamed oral lesions like RAU.

Medicinal cannabis products are defined as dried female flowers of *Cannabis sativa* or *Cannabis indica* whose CBD concentration range from 0.1–19% [19]. Cannabis side effects are dose-dependent. Most adverse events of cannabis can be mitigated using a “start low and go slow” dosing strategy [20]. One study assessed the safety and preventive effects of 0.1% CBD on chemotherapy-induced peripheral neuropathy (CIPN) in patients receiving oxaliplatin- or paclitaxel-based chemotherapy, and concluded that CBD reduced the early symptoms of CIPN without major adverse events [21].

The safety of CBD has been demonstrated in animal and human studies. It has a low adverse effect profile, including when chronically used [22]. However, there are some reports of sedation and somnolence with CBD use. Prescribing CBD with other sedative drugs, such as opioids and benzodiazepines, can cause severe respiratory depression. Contraindications to CBD administration include allergies to cannabidiol (signs of cutaneous irritation or anaphylactic reaction), history of drug or alcohol addiction (due to CBD’s addictive properties), and history of mood disorders, depression, or suicidal thoughts (due to a correlation with increased suicidal thoughts and CBD use) [23].

CBD might be an alternative approach for RAU management due to its known clinical benefits, particularly in reducing pain and inflammation and promoting wound healing [18]. However, topical 0.1% CBD has not been assessed for the risk for skin allergy, local and systemic side effects on normal oral mucosa, and for the clinical efficacy in treating RAU. Hence, the objectives of this study were: (1) to investigate the risk for allergic skin reactions to topical 0.1% CBD, (2) to assess the local and systemic side effects of topical CBD on normal oral mucosa, and (3) to evaluate the clinical efficacy of topical CBD for managing RAU.

## Methods

### CBD preparation

A 5%w/w CBD (CBD-5CC) extract was obtained from *Cannabis sativa* and donated by Leapdelab Co., Ltd., Samut Prakan, Thailand*.* CBD oral pastes were prepared by the Faculty of Pharmaceutical Sciences, Chulalongkorn University, Bangkok, Thailand, and passed in vitro and animal safety screening.

### Study design and sample

The clinical phases of this study were conducted at the Oral Medicine Clinic, Faculty of Dentistry, Chulalongkorn University, Bangkok, Thailand and performed with informed consent following protocols approved by the Human Research Ethics Committee of the Faculty of Dentistry, Chulalongkorn University (certificate number HREC-DCU 2021–048 approved on 9 July 2021). Informed consent was obtained from all subjects. All methods were performed in accordance with the Declaration of Helsinki.

### Phase 1: CBD patch test outcomes on human skin

To investigate the possible allergic reactions on the skin (e.g., contact dermatitis) while using CBD oral paste, 100 healthy subjects (50 males and 50 females, 18–65 years old) were recruited to participate in this study. 0.1% CBD was loaded in four Finn Chambers (Epitest, Tuusula, Finland) and placebo (pure oral paste) was loaded in four other chambers. The chambers were applied to the subjects’ skin on the upper back. After 48 h, the chambers were removed and 15 min later, any allergic reaction was scored according to the International Contact Dermatitis Research Group (ICDRG) standards [24]. Scoring was performed again 24 h later. The subjects and examiner (CU) were blinded to which chambers contained CBD. The examiner did the relabeling for statistical analysis.

### Phase 2: CBD safety clinical outcomes after application on normal oral mucosa

To assess the local and systemic side effects of CBD oral paste after applying it on healthy oral mucosa, 50 healthy subjects (25 males and 25 females, 18–65 years old) were recruited to participate in this study. The subjects were instructed to apply CBD over their lower labial mucosa with a provided calibrated spoon 3 times/day after meals for 7 d. Oral examination, vital signs, and blood tests were performed before and after CBD use. The blood parameters evaluated comprised glucose, hematocrit, sodium, potassium, chloride, total CO_2_, serum glutamic oxaloacetic transaminase, serum glutamic pyruvic transaminase, alkaline phosphatase, total protein, total bilirubin, albumin, blood urea nitrogen, and creatinine.

### Phase 3: CBD efficacy outcomes in subjects with RAU

#### Study design

A randomized parallel double-blind controlled trial design was performed in phase 3. To measure the efficacy of CBD for treating RAU, 72 subjects with RAU randomly received one of three topical interventions: 0.1% CBD, 0.1% triamcinolone acetonide (TA), or placebo using simple randomization according to a manually generated list of random numbers with a 1:1:1 allocation ratio.

#### Sample size

The sample size was calculated using the G*Power program version 3.1.9.7 for 80% power, 95% confidence level, and 0.4 effect size according to the range of ulcer sizes reported in a previous study [25]. The final estimated total sample size was 60. To compensate for error or loss of subjects during follow-up, based on an anticipated drop-out rate of 15%, a total sample size of 72 subjects were recruited.

#### Subjects

The subjects fulfilled the following inclusion criteria: age between 18–65 years old, willing to participate and provide informed consent, and had a history of RAU (at least 2 times/year) on nonkeratinized oral mucosa, presenting with 1–3 minor aphthous ulcers (of ≤ 48 h duration) that were 2–10 mm in diameter and easily accessible for evaluation. The exclusion criteria for phase 3 comprised: history of allergy to CBD, pregnancy/lactation, concurrent oral bacterial/fungal/viral infections, ulcers as a manifestation of a systemic disease, e.g., Crohn’s disease, Behcet’s disease, or anemia, ulcers from trauma, diabetes mellitus patients, treatment with systemic steroids, oral retinoids, or other immunomodulatory agents within 1 week, treatment with acetaminophen, nonsteroidal anti-inflammatory drugs, or oral topical medications within 48 h or during project participation, history of dental surgery within 2 weeks of entering the study, or orthodontic braces or retainers that might come in contact with the ulcer.

#### Allocation concealment and blinding

The pharmacological compounds were sealed in sequentially numbered identical containers. The placebo was matched to the CBD oral paste for taste, color, appearance, and smell. A research assistant (WK) generated the random allocation sequence, enrolled the subjects, and assigned the subjects to the interventions. The subjects and investigator (CU) were blinded to the type of intervention.

#### Interventions

The subjects were randomly placed into one of the three interventions: 0.1% CBD, 0.1% TA, or placebo. Seventy-two subjects (24 subjects for each intervention) were enrolled in this study. The interventions were applied with a provided calibrated spoon to the ulcers 3 times/day after meals for 7 days. When the subjects developed RAU more than one time (at least 2 weeks apart), they could reenter the study, and received a different topical intervention. Each subject was interviewed at each visit by the same investigator regarding the emergence of any adverse reactions. The ulcers were diagnosed by an oral medicine specialist. If there was more than one ulcer, the ulcer with the easiest access was selected for investigation.

#### Outcomes

##### Ulcer severity score (USS)

The USS is indicative of the disease severity. This score incorporated six ulcer characteristics: number, size, duration, ulcer-free period, site, and pain [26]. It was used at the first visit as baseline.

##### Ulcer size

The ulcer size was measured on day 0, 2, 5, and 7. Two ulcer size parameters consisting of the pseudomembranous ulcer size and erythematous border size were measured. The ulcer diameters were measured using a calibrated dental probe with millimeter markings, and the ulcer sizes were calculated using formulas for the surface area of a circle or ellipse. The ulcers were photographed alongside a visual reference of known size, and a researcher drew the boundary of the pseudomembranous ulcer and erythematous border size on the captured image. The images were analyzed using computer software (Image-Pro Plus version 4.5 for Windows, Media Cybernetics, Rockville, MD, USA).

##### Daily pain ratings

Pain ratings using a visual analog scale (VAS) consisting of a 100-mm horizontal line between the endings marked “no pain” and “unbearable pain” were recorded daily by the subjects.

##### Subject satisfaction

On the last day, the subjects rated their satisfaction with the intervention used on a scale of 0 (not satisfactory) to 10 (the most satisfactory). The subjects who used all three topical interventions were asked to rank the interventions according to their preference (from most to least preferred).

##### QoL improvement

The subjects completed a QoL questionnaire using the Thai Oral Health Impact Profile-14 (OHIP-14) at the first and last visit.

### Statistical analysis

The background and demographic data were summarized using descriptive statistics. In phase 2, the normal distribution for each variable was determined using the Shapiro–Wilk test. Matched paired differences of vital signs and blood tests before and after CBD use were analyzed using the paired t-test (normally distributed variables) or the Wilcoxon signed-rank test (not normally distributed variables). In phase 3, the normal distribution for each variable was determined by the Kolmogorov–Smirnov test. Group differences among the three interventions were compared using one-way ANOVA followed by the Bonferroni post hoc test (normally distributed variables) or the Kruskal–Wallis test/Median test followed by Bonferroni correction for multiple tests (not normally distributed variables) for pseudomembranous ulcer size, erythematous border size, pain level, satisfaction, and OHIP-14 score at each monitoring point. The OHIP-14 scores at the first and last visit in each group were compared using the paired t-test (normally distributed variables) or the Wilcoxon signed-rank test (not normally distributed variables). The data were analyzed using SPSS software (SPSS 28 for Windows; SPSS, Chicago, IL, USA). A *p*-value of ≤ 0.05 was considered significant.

## Results

### Phase 1: CBD patch test outcomes on human skin

None of the subjects exhibited allergic reactions (signs of erythema, vesicles, or ulceration) or contact dermatitis on any CBD-treated area.

### Phase 2: CBD safety clinical outcomes after application on normal oral mucosa

None of the subjects experienced or reported an obvious adverse reaction (irritant response or allergic reaction) on their oral mucosa. Furthermore, no anaphylactic reactions were observed in the respiratory or circulatory system and no signs of liver dysfunction were reported (e.g., nausea, vomiting, jaundice, right upper quadrant pain, or dark urine). The subjects’ vital signs and blood parameters were stable before and after the 7-day CBD intervention (*p* > 0.05).

### Phase 3: CBD efficacy outcomes in subjects with RAU

**Fig. 1:**
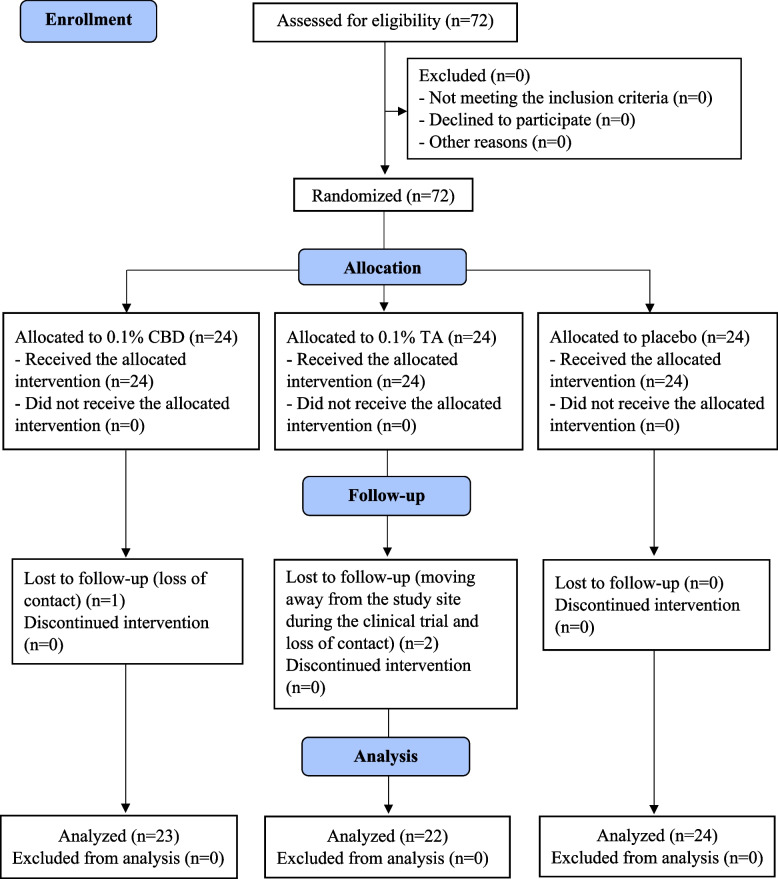
A diagram of the participant flow in phase 3 according to CONSORT guidelines

Seventy-two patients were enrolled in this study during September 2021–April 2022. Twenty-four subjects were assigned to each intervention. One and two subjects were lost to follow-up in the CBD and TA group, respectively. Recruitment was stopped once the sample size was reached due to the study timelines and budget constraints. A diagram of the participant flow in this phase according to the Consolidated Standards of Reporting Trials (CONSORT) guidelines is presented in Fig. 1. There were no differences in the demographics or ulcer histories, except for ulcer duration, between the three intervention groups (Table 1), (*p* = 0.039). However, after adjusting the data using the Bonferroni correction for multiple tests, the difference was no longer significant.

**Table 1:** Baseline data of the study participants

	**CBD (*n* = 23)**	**TA (*n* = 22)**	**Placebo (*n* = 24)**	***p***
Age (years), mean (SD)	36.7 (11.3)	35.9 (10.4)	33.0 (10.1)	0.410^a^
Sex				
Male, *n* (%)	2 (2.9)	4 (5.8)	7 (10.2)	0.199^b^
Female, *n* (%)	21 (30.4)	18 (26.1)	17 (24.6)	
Duration of the ulcer (hours), mean (SD)	42.8 (10.5)	35.1 (13.6)	34.7 (13.0)	0.039^a,c^
Pseudomembranous ulcer size on day 0 from the photograph (mm^2^), mean (SD)	6.0 (3.9)	7.2 (4.9)	6.5 (5.0)	0.755^a^
Erythematous border size on day 0 from the photograph (mm^2^), mean (SD)	15.3 (8.9)	17.7 (11.9)	19.0 (20.3)	0.876^a^
VAS on day 0 (mm), mean (SD)	53.7 (19.6)	50.2 (23.7)	53.2 (25.7)	0.861^d^
USS (scores), mean (SD)	16.3 (4.3)	17.6 (4.6)	19.3 (4.1)	0.066^d^
OHIP-14 score at the first visit (scores), mean (SD)	25.1 (9.4)	24.7 (9.8)	30.5 (11.9)	0.110^d^

^a^*p*-Values from the Kruskal–Wallis test

^b^*p*-Values from the Pearson’s chi-square test

^c^After adjusting the data with the Bonferroni correction for multiple tests, the difference was no longer significant (placebo-TA: *p* = 1.000, placebo-CBD: *p* = 0.070, TA-CBD: *p* = 0.099)

^d^*p*-Values from one-way ANOVA

### Ulcer size reduction

**Fig. 2:**
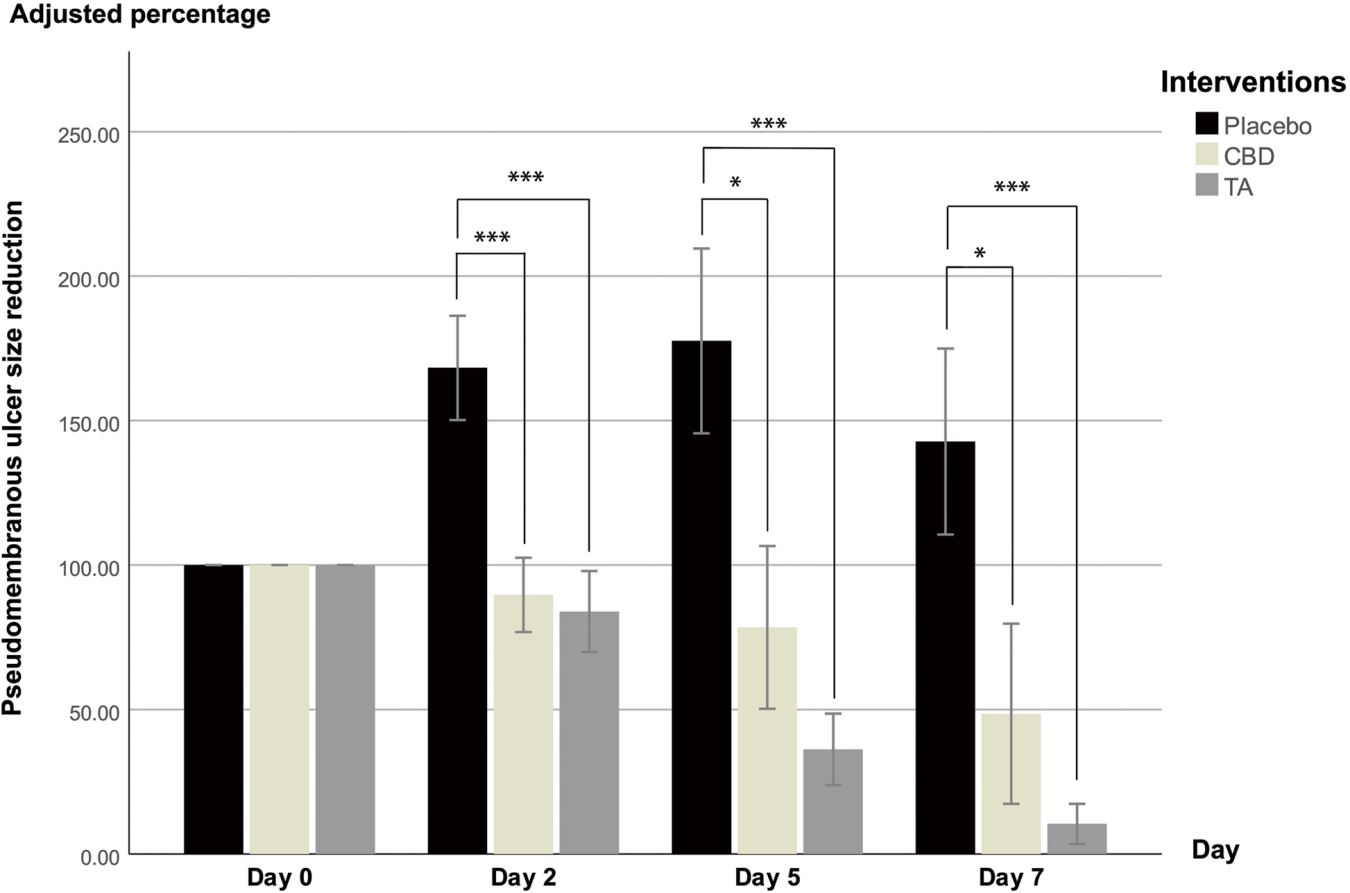
Pseudomembranous ulcer size reduction. The Y-axis values represent percentages. Error bars display the standard error of the mean (SEM). Significance is portrayed as * *p* ≤ 0.05, ** *p* ≤ 0.01, and *** *p* ≤ 0.001

**Fig. 3:**
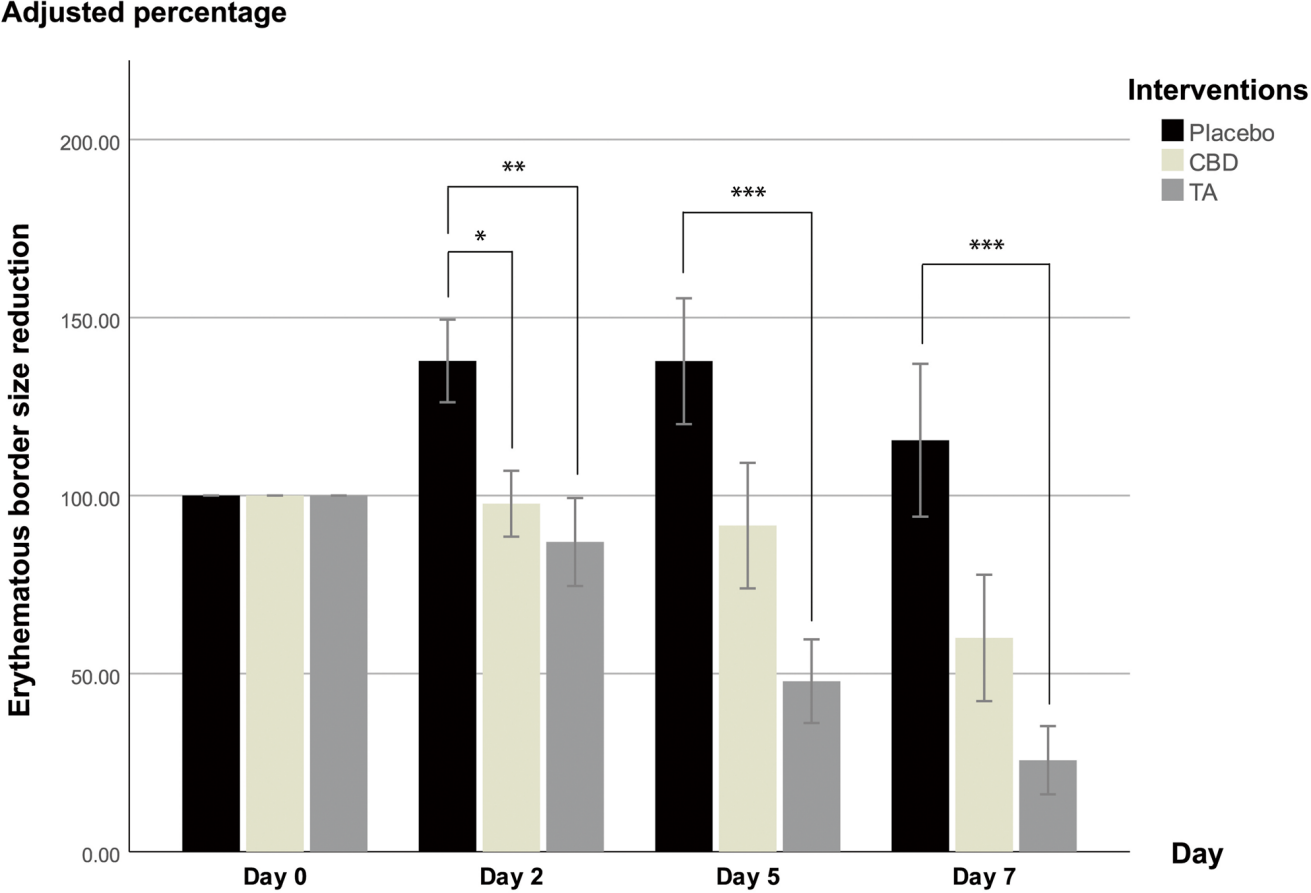
Erythematous border size reduction. The Y-axis values represent percentages. Error bars display the SEM. Significance is portrayed as * p ≤ 0.05, ** *p* ≤ 0.01, and *** *p* ≤ 0.001

The ulcer size was adjusted to a percentage compared with baseline (100%). The ulcer size reduction analysis among the three interventions indicated that the pseudomembranous ulcer size was almost 100% smaller in the CBD group on day 5 (Fig. 2) and the erythematous border size was 40% smaller in the CBD group on day 2 (Fig. 3) compared with the placebo group. The average ulcer size in the placebo group increased approximately 175% and 140% on day 5 and 7, respectively, compared with baseline. CBD and TA reduced the pseudomembranous ulcer and erythematous border size from day 2 onwards. In contrast, the placebo markedly increased the pseudomembranous ulcer and erythematous border size on day 2 and 5, however, these sizes were decreased on day 7. The statistical analysis revealed that CBD and TA significantly reduced the pseudomembranous ulcer size more than placebo at all monitoring points (*p* < 0.05). CBD reduced the erythematous border size greater than placebo only on day 2 (*p* = 0.042). In contrast, the erythematous border size reduction in the TA group was greater than the placebo group at all monitoring points (*p* < 0.05). Although CBD reduced the pseudomembranous ulcer and erythematous border size less than TA, the differences were not significant.

The pseudomembranous ulcer and erythematous border size after treatment with placebo (representing spontaneous remission) were larger than before treatment, whereas CBD and TA continually decreased the ulcer size at every monitoring point. Thus, CBD and TA accelerated ulcer healing compared with natural healing.

### Daily pain ratings

**Fig. 4:**
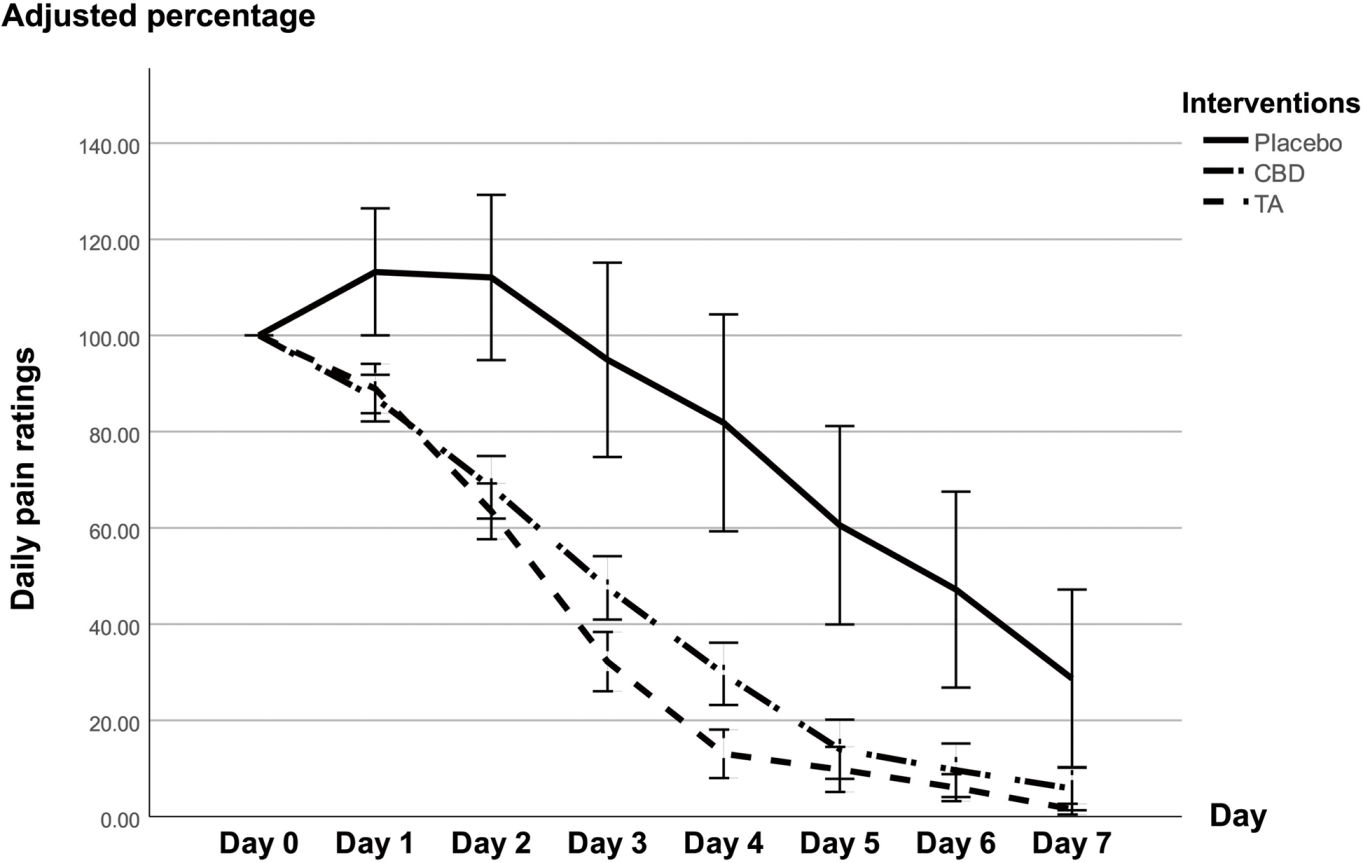
Daily pain ratings. The Y-axis values represent percentages. Error bars display the SEM

The ulcer pain scores (VAS) were adjusted to a percentage compared with baseline (100%). Comparing the daily pain ratings between the three interventions, topical CBD and TA decreased the pain levels from day 1 onwards, while the placebo markedly increased the pain levels on day 1–2 and then gradually decreased the pain levels from day 3 onwards, as demonstrated in Fig. 4. Statistical analysis revealed that TA significantly reduced the pain levels on day 4 (*p* = 0.009), day 5 (*p* = 0.023), and day 7 (*p* = 0.008) greater than placebo, whereas the pain levels in the CBD group were lower than the placebo group only on day 5 (*p* = 0.039). However, there were no significant differences in pain reduction between the CBD and TA interventions.

RAUs typically become larger and more painful over the first few days of their development and then gradually spontaneously heal in 4–14 days [7]. Although some subjects who received placebo still had large ulcers on day 5 and 7, the average pain ratings in the placebo group were lower than baseline from day 3 onwards, while CBD and TA reduced the pain levels from day 1 onwards. The pain ratings for the three interventions decreased over time.

### Subject satisfaction with topical interventions

The subjects who received the TA and CBD topical interventions were more satisfied with the intervention (average satisfaction score of 8.32 vs. 7.48, respectively,* p* > 0.05), compared with placebo (average score of 6.17, *p* = 0.025 compared with TA). However, the differences in the subject’s satisfaction between the CBD and TA interventions were comparable.

At the end of the study, four subjects had been treated with all three interventions. When these subjects ranked the interventions in order of preference, three subjects (75%) selected TA as the most preferred intervention followed by CBD, and placebo was the least preferred. One subject (25%) picked CBD as the most preferred intervention followed by TA and placebo.

### QoL improvement

The statistical analysis of the OHIP-14 score reduction in each group revealed that the three interventions significantly reduced the OHIP-14 scores between first and last visit (*p* < 0.001). Higher scores correspond to a poorer oral QoL. The QoL improvement results demonstrated that the subjects who received CBD reported the greatest reduction in OHIP-14 scores with an average delta score of 19.83, followed by TA (19.59), and placebo (17.71). However, the delta score in the OHIP-14 between the first and last visit was relatively similar among the three interventions (*p* = 0.831). The subjects’ pain, functional limitation, and discomfort resolved over time because RAU is a self-limiting ulceration [2].

## Discussion

In recent years, complementary and alternative medicine (CAM) has been increasingly used worldwide. CAM is defined as a group of diverse medical and health care systems, practices, and products that are not presently considered to be part of conventional medicine [27]. Herbal medicines were the most frequently used type of CAM [28]. To minimize the side effects of synthetic drugs, herbal medicines have been advocated as an alternative for treating various diseases. Several publications have endorsed the medicinal effects of herbal medicines. The use of pomegranate seed powder, a fruit in the Lythraceae family, decreased fasting blood glucose and glycated hemoglobin in patients with type 2 diabetes mellitus [29]. Herbal compresses for maternal breast engorgement during postpartum reduced breast engorgement pain greater than hot compresses due to the analgesic and anti-inflammatory effects of the herbal ingredients [30]. In dentistry, plant-based drugs, such as aloe vera and curcumin, were shown to be effective and safe drugs for RAU and oral mucositis due to their anti-inflammatory effects [31]. Currently, CAM is gaining popularity and has attracted the attention of researchers and patients as a complementary treatment for managing numerous diseases.

The present study evaluated the use of 0.1% CBD as a topical treatment for RAU. None of the subjects experienced an allergic reaction to 0.1% CBD either on their skin or oral mucosa. Applying CBD to normal oral mucosa for 7 days did not affect the subjects’ vital signs, glucose, hematocrit, electrolyte levels, or liver and kidney function. These results indicate that topical 0.1% CBD is safe for human skin and oral mucosa application.

In this study, phase 3 was conducted as a randomized controlled trial (RCT) in which ulcer size, erythematous border size, pain level, satisfaction, and OHIP-14 score were evaluated. These are the standard variables or factors that are often assessed when evaluating the pharmacological efficacy for RAU management. The findings from the present randomized, double-blind controlled clinical trial study indicate that CBD treatment reduced pseudomembranous ulcer and erythematous border size and alleviated pain during the 7-day application. The pseudomembranous ulcer size was significantly reduced due to CBD’s wound healing promotion and anti-inflammatory effects [18]. The erythematous circumscribed border represents the level of inflammation [32]. CBD reduced the erythematous border size greater than placebo on day 2, similar to a study using CBD in oral ulcer lesions in rats, which determined that CBD exerts an anti-inflammatory effect in the early phase of wound healing [33].

The pain scores of the three intervention groups decreased over time [25] because RAU is a self-limiting ulcer [2]. Thus, some subjects who received placebo might have felt better from day 3 onwards due to the reduced ulcer size. At the end of the study, some subjects who received placebo still had large ulcers, however, the average daily pain scores on day 3–7 from this group were lower than baseline. The pain relief effect of the placebo may stem from the paste layer that protects the ulcer from physical and chemical stimuli. Interviewing the two groups of subjects that received the oral paste alone revealed that it produced a lingering cool effect on the ulcer. Moreover, the subjects were blinded to the intervention type, and the placebo may have produced some psychological effects [25].

A study revealed that RAUs affect patient QoL due to pain (during talking, eating, and swallowing), discomfort (impaired food and liquid intake), interpersonal relationship problems, and self-confidence [34]. The higher OHIP-14 scores at the first visit in our study confirmed that RAUs influence an individual’s QoL. Although the QoL scores measured using the Thai OHIP-14 between the first and last visit in each group were significantly reduced, the difference in the reduced OHIP-14 scores among the three interventions was similar because RAUs resolve over time [2].

A previous study [25] suggested using a dental probe for measuring ulcer size. In the present study, we measured the ulcer size with a calibrated dental probe and captured images with a visual reference. Although the ulcer diameters measured using a dental probe were calculated using formulas for the surface area of a circle or ellipse, the exact ulcer size was difficult to calculate due to the imperfectly round or ovoid ulcers and could have resulted in inaccurate ulcer sizes. We compared the ulcer size between the measurements obtained using a dental probe and photograph; the results revealed that they were significantly different. Therefore, we decided to exclude the ulcer size data measured with a dental probe. A minor error for the measuring method using image analysis could stem from the difference in pulling forces used to retract the oral mucosa. To minimize this inaccuracy, we always asked the subjects to retract their oral mucosa with similar pulling forces. The photographs were also taken at the same position at every monitoring point.

This study has some limitations that must be carefully considered. One limitation was the two-dimensional measurement of the ulcer. Because ulcer size reduction and pain relief are not the only signs of improvement in healing ulcers, decreases in ulcer depth should also be measured. If the ulcer depth variable is incorporated into the ulcer healing outcomes, the findings may be more comprehensive. However, USS was used at baseline and some parts of the USS overlapped with other measurements, i.e., ulcer size and pain score, that were recorded at each monitoring visit; and the ulcer numbers and sites were collected at baseline. A comprehensive score that includes QoL outcomes for assessing improvement rates during RAU treatment interventions would be relevant to use in further clinical investigations. Furthermore, topical CBD dosages above 0.1% have not been investigated in early trials and should be considered in future RCTs for RAU management to improve the clinical efficacy while comparing CBD side by side with TA.

To our knowledge, this is the first randomized clinical trial investigating the clinical effects of 0.1% CBD for RAU topical treatment. The efficacy of CBD was clinically meaningful, specifically on reducing the pseudomembranous ulcer and erythematous border size and on pain relief.

## Conclusions

This clinical study demonstrated that topical 0.1% CBD reduced ulcer size and accelerated ulcer healing without any reported local (signs of allergic and anaphylactic reactions) or systemic (vital sign and blood test alteration) side effects. Furthermore, in the RCT, topical CBD exerted an anti-inflammatory effect by reducing the erythematous border size in the early stage and decreased pain intensity in the late stage of RAU. Thus, CBD may be appropriate for RAU patients who choose not to take topical steroids, except for cases where CBD is contraindicated, such as being allergic to CBD, a history of drug or alcohol addiction, and a history of mood disorders.

## Data Availability

The datasets used and/or analyzed during the current study are available from the corresponding author on reasonable request.

## Abbreviations

RAU: Recurrent aphthous ulcer
CBD: Cannabidiol
TA: Triamcinolone acetonide
USS: Ulcer severity score
VAS: Visual analog scale
QoL: Quality of life
OHIP-14: Oral health impact profile-14
SD: Standard deviation
SEM: Standard error of the mean
RCT: Randomized controlled trial
CAM: Complementary and alternative medicine
